# Cannabis-Induced Acute Encephalopathy in a 94-Year-Old Woman Due to Family Administration of Cannabidiol (CBD) Products: A Case Report

**DOI:** 10.7759/cureus.37927

**Published:** 2023-04-21

**Authors:** Mohamad K Moussa, Mary Ann Kirkconnell Hall, Joyce Akwe

**Affiliations:** 1 Division of Hospital Medicine, Emory University School of Medicine, Atlanta, USA; 2 Hospital Medicine, Emory Johns Creek Hospital, Johns Creek, USA; 3 Hospital Medicine, Atlanta Veterans Affairs Medical Center, Atlanta, USA

**Keywords:** elderly cannabis use, older adults cannabis use, encephalopathy, unintentional cannabis ingestion, elderly individuals, tetrahydrocannabinol (thc), cannabidiol (cbd), cannabis oil

## Abstract

In the United States, cannabis use is rising, including among older adults, as is unintentional ingestion. We describe the case of a 94-year-old woman admitted with altered mental status, diarrhea, and hallucinations. She lived with her family, who had noticed recent confusion with weakness, poor oral intake, and loose bowel movements.

In the emergency room, her vital signs revealed mild tachycardia and hypotension. She was lethargic, disoriented, confused, and anxious but could answer simple questions. The attending hospitalist administered the Mini-Cog dementia screening and found that the patient was oriented to herself only and was unable to perform word recall tests or complete a clock drawing. The rest of her physical exam was within normal limits for her age.

Despite a workup including urine culture, chest X-ray, and computed tomography scan of her head, no organic source for her mental change was found. After five days of admission, a close relative confessed that they had given the patient cannabis oil (marketed as “pure CBD,” i.e., cannabidiol, a nonpsychoactive cannabis derivative widely touted as a remedy for pain, anxiety, and anorexia) in the form of edible brownies to help her with her persistent back pain and poor appetite. We performed a urine drug screen for tetrahydrocannabinol (THC), the psychoactive component of cannabis, which verified cannabis use, as well as exposure to THC. The patient recovered to baseline with supportive care.

Currently, there is no governing body or framework for the regulation of cannabis products in the United States. Nonprescription CBD products are not regulated by the US Food and Drug Administration, and these products are not tested for safety, efficacy, or quality. Some producers voluntarily conduct such testing, but there is no regulatory oversight, and consumers may be unaware of the need for testing and/or which testing bodies are credible. Given the rapidly increasing proportion of older adults who are cannabis users, physicians should inquire about outpatient use of cannabis in general and CBD in particular during discussions with patients, even the most elderly.

## Introduction

In the United States, cannabis use is rising, including among older adults [1,2]. From 2006 to 2013, past-year cannabis use increased by 57.8% in adults ages 50-64 and by 250% in those aged 65 and older [3]. Cannabis is by far the most frequently used illicit substance worldwide, and older adults are one of the fastest growing user groups [4]. According to a 2020 study, cannabis use among adults aged 65 and older increased from 2.4% in 2015 to 4.2% in 2018 in the United States [5]. 

Unintentional ingestion is also rising as products with cannabis for multiple uses become legal or decriminalized [6-8]. In 2018, the Farm Bill (i.e., the US federal government's primary agricultural policy and regulatory legislation, revised every five years) federally legalized cannabidiol (CBD) products containing a concentration of less than 0.3% delta-9-tetrahydrocannabinol (THC), the psychoactive component of cannabis. The CBD-based drug Epidiolex has received US Food and Drug Administration (FDA) approval for the treatment of some seizure and epilepsy syndromes, and several synthetic THC cannabinoid medications (Marinol, Cesamet, and Syndros) have been approved for use as therapeutic agents for nausea associated with chemotherapy and loss of appetite/anorexia among AIDS patients [9].

Both CBD and THC are derived from the *Cannabis sativa* plant. Many nonprescription cannabis products, particularly those marketed for recreational use, contain THC. A variety of products contain both active components of cannabis [9]. Products that purportedly contain only CBD are sold for a variety of causes (including pain, insomnia, and anxiety) and are claimed to be nonintoxicating [10]. Unlike THC, CBD is not generally associated with accounts of psychosis, anxiety, or memory impairment. However, a recent report by the FDA found that most CBD products do not contain the amount of CBD claimed by their labels, and while the majority contained trace amounts of THC below the 0.3% threshold, several contained larger amounts, up to as much as 1 mg/serving [11].

## Case presentation

A 94-year-old female was admitted to the hospital with three days of altered mental status, diarrhea, and hallucinations. She lived with her family, who had noticed that she had recently seemed more confused and weaker than usual, had poor oral intake and loose bowel movements, and was experiencing worsening of chronic back pain attributed to osteoporosis and arthritis. According to her family, prior to the preceding three days, she was mentally “sharp” with no confusion or hallucination, and she had no previous episodes of loose stools. Her only existing complaints prior to the onset of confusion, hallucinations, and diarrhea were poor appetite and low back pain.

The patient did not take any prescription medications. She denied any sick contact, taking a stool softener or laxatives, or having a recent fall. Her family corroborated that she had no recent falls, food poisoning, or other exposures to which her symptoms could be attributed.

In the emergency room, her vital signs revealed mild tachycardia with a heart rate of 105 beats per minute and hypotension with a blood pressure of 90/65 mmHg associated with dry oropharynx and dry skin turgor. Her initial chemistry lab revealed mild hyponatremia of 127 mEq/L, creatinine of 1.4 (baseline 0.9-1.0), and mild acidosis with mildly elevated liver function test results. Her complete blood count was normal.

On the neurologic exam, she was lethargic, disoriented, confused, and with obvious anxiety (likely due to hallucinations she was experiencing) but could answer simple questions. The attending hospitalist administered the Mini-Cog screening assessment and found that the patient was oriented to herself only (oriented to ×1) and was unable to perform word recall tests or to complete a clock drawing. Her reflexes were depressed, and she had decreased muscle tone in her lower extremities and mild tremor. The rest of her physical exam was within normal limits for her age.

Due to clinical and laboratory evidence of significant dehydration, likely precipitated by poor intake of fluid caused by sedation, she was started on intravenous (IV) fluids, given one dose of IV ceftriaxone for possible urinary tract infection and altered mental state, and admitted to the hospital medicine service for follow-up.

On the floor, the patient started to have worsening confusion, especially during the late afternoon (suspected “sundowning” or delirium). Her family stayed with her to help with orientation.

Our initial approach was to look for an organic source for her mental change. Her laboratory results showed mild dehydration that was corrected with IV hydration. We noted mild pyuria (40 white blood cells and eight red blood cells per high power field) and started her on IV ceftriaxone 1 gm/day. Her urine culture was negative - there were few bacteria noted with no leukocyte esterase or nitrite - so the antibiotics were stopped. A computed tomography (CT) scan of her head showed moderate chronic ischemic microvascular disease with mild dilatation of ventricles, possibly reflecting mild hydrocephalus; this chronic white matter disease was appropriate for her age. A chest X-ray found no acute findings. No other infectious or metabolic causes were found. After two days of supportive care, her initially depressed reflexes and mild tremors resolved, but her confusion and agitation remained. Her initial diagnosis was a delirium of unknown origin. 

After five days of admission, during which her condition remained stable, a family member confessed that they had given the patient cannabis oil (marketed as “pure CBD”) in the form of edible brownies to help her with her persistent back pain and poor appetite. To confirm this, we performed a urine drug screen for THC that verified cannabis use, as well as exposure to the psychoactive compound.

The patient started to feel better with supportive care (IV fluid, an IV multivitamin, and topical lidocaine to help with her low back pain).

The patient stayed at the hospital for six days and was discharged home with home health physical and occupational therapy support and a presumed diagnosis of cannabis-induced acute encephalopathy. She had returned to her baseline level of cognitive function by the time of discharge. Her family promised not to give her any materials containing cannabis or cannabis derivatives due to the undesirable outcomes she had experienced.

## Discussion

Our patient presented with what we determined were acute signs of intoxication from cannabis use, specifically THC. We believe that the patient developed such a disabling level of cannabis intoxication because she was already dehydrated from poor oral intake before ingestion of THC-containing brownies. This intoxication led to delirium and diarrhea, which in turn caused hypotension, acute kidney injury (AKI), and mild elevation of liver function tests.

We believe that her hypotension, hyponatremia, and AKI were the results rather than the cause of her delirium and encephalopathy; all of these problems except her delirium and hallucinations improved with IV hydration. While the differential diagnosis of delirium of other causes is possible given the patient’s age (94 years) with limited reserves, the development of delirium immediately following cannabis ingestion that resolved fully with supportive care is almost certainly due to exposure to high levels of THC.

Clinical manifestations of THC exposure include physiologic signs such as tachycardia, blood pressure changes, conjunctival injection, dry mouth, slowed or slurred speech, and ataxia [12]. Psychological manifestations include euphoria and relaxation, or conversely anxiety and paranoia or feelings of unreality [6], as well as sedation and increased appetite [12]. Cannabis use acutely impairs neuropsychological functions, especially attention, concentration, episodic memory, and associative learning [13]. Depression, anxiety (including panic attacks), perceptual disturbances, and changes in thought content such as transient paranoia or frank psychosis are not uncommon [13].

Currently, there is no governing body or framework for the regulation of cannabis products. Nonprescription CBD products are not regulated by the FDA, and these products are not required to be tested for safety, efficacy, or quality. Some producers voluntarily conduct such testing, but there is no regulatory oversight, and consumers may be unaware of the need for testing and/or which testing bodies are credible. Some over-the-counter preparations contain much higher or lower quantities of CBD, and many products are mislabeled: even products labeled as pure CBD may also contain significant quantities of the psychoactive compound THC [11], as did the oil our patient’s family member used to prepare her brownies.

Patients and families use over-the-counter CBD products for a wide array of symptoms, risking potential exposure to THC. THC’s adverse effects - drowsiness, dizziness, fatigue, agitation, hallucinations, and even diarrhea [1,2,14] - can have detrimental effects on frail elderly individuals [15], increasing already elevated risks of falls [14] and dehydration, and have the potential to be mistakenly managed as signs of acute infection or dementia rather than intoxication. Cannabis compounds are sold as an additive in common consumable products such as cookies, candies, or healthy snacks, facilitating unintentional ingestion or ingestion of larger amounts than intended [6,7].

The medical literature on cannabis use in the elderly is growing. A recent scoping review [16] found that the risk/benefit ratio of medical cannabis in older adults was unclear, in light of associations observed between cannabis use and illicit substance abuse, poor mental health, and increased healthcare utilization in this population. While prescription CBD is a demonstrably effective anticonvulsant [9], the evidence for cannabis’ effectiveness in reducing chronic or acute pain is equivocal [17]. A recent meta-analysis of CBD use [18] found few adverse effects, most commonly diarrhea, but also nausea, drowsiness, fatigue, dry mouth, and mood changes. Others have noted CBD interactions with medications [15] including antidepressants, opioids, antiepileptic drugs [19], anticoagulants and antiplatelet medications [20], and acetaminophen and alcohol [19].

Given the rapidly increasing proportion of older adults who are cannabis users [3,5], physicians and other health care providers should inquire about outpatient use of cannabis in general and CBD in particular during discussions with patients, even the most elderly.

## Conclusions

There is abundant evidence in the literature and medical practice that more and more people, including older adults, are using cannabis products for medical and/or recreational purposes. As cannabis-containing products become more widely available and their use destigmatized, a correspondingly growing number may unintentionally ingest them or consume unintentionally large amounts. The effects of these products on the elderly have not been well documented, especially in association with the comorbidities that are more prevalent in this age group. More research is needed to fully understand the potential benefits and risks of cannabis use in the elderly and to develop guidelines for the safe and effective use of various cannabis products and derivatives in this vulnerable population.
